# Functional Proteomic Profiling of Phosphodiesterases Using SeraFILE Separations Platform

**DOI:** 10.1155/2012/515372

**Published:** 2012-11-25

**Authors:** Amita R. Oka, Matthew P. Kuruc, Ketan M. Gujarathi, Swapan Roy

**Affiliations:** ProFACT Proteomics Inc., 1 Deer Park Drive, Suite M, Monmouth Junction, NJ 08852, USA

## Abstract

Functional proteomic profiling can help identify targets for disease diagnosis and therapy. Available methods are limited by the inability to profile many functional properties measured by enzymes kinetics. The functional proteomic profiling approach proposed here seeks to overcome such limitations. It begins with surface-based proteome separations of tissue/cell-line extracts, using SeraFILE, a proprietary protein separations platform. Enzyme kinetic properties of resulting subproteomes are then characterized, and the data integrated into proteomic profiles. As a model, SeraFILE-derived subproteomes of cyclic nucleotide-hydrolyzing phosphodiesterases (PDEs) from bovine brain homogenate (BBH) and rat brain homogenate (RBH) were characterized for cAMP hydrolysis activity in the presence (challenge condition) and absence of cGMP. Functional profiles of RBH and BBH were compiled from the enzyme activity response to the challenge condition in each of the respective subproteomes. Intersample analysis showed that comparable profiles differed in only a few data points, and that distinctive subproteomes can be generated from comparable tissue samples from different animals. These results demonstrate that the proposed methods provide a means to simplify intersample differences, and to localize proteins attributable to sample-specific responses. It can be potentially applied for disease and nondisease sample comparison in biomarker discovery and drug discovery profiling.

## 1. Introduction

Proteomic profiling based on enzyme activity is assuming significance in drug discovery as it becomes possible to profile selectivity of drugs and their mechanism of action [1]. Such an approach focuses on protein function, an aspect which has been missing from expression proteomics [1]. A functional proteomic profiling approach has the potential not only to help identify targets for diagnosis and therapy [2], specifically in personal medicine [3, 4], but also to reveal the underlying mechanisms of action of disease-sustaining proteins [5].

Methods for global analysis of protein expression and function, including liquid chromatography with mass spectrometry (MS) for shotgun analysis [6, 7], yeast two-hybrid methods [8], and protein microarrays [9], have been crucial in developing the field of proteomics, but they do not provide an accurate assessment of functional states of proteins in cells and tissues [10]. Activity-based protein profiling (ABPP) was first demonstrated for serine hydrolyses [11] and has now been applied to other enzyme classes such as kinases, phosphatases, and histone deacetylates [10, 12]. ABPP typically uses active site-directed covalent probes to interrogate specific subsets (families) of enzymes in complex proteomes to provide a quantitative assessment of the functional state of individual enzymes in the family [10]. The probe-bound enzymes can be visualized with SDS-PAGE or purified using affinity tools for peptide or labeling site identification with MS [10]. Although this approach is promising, it is limited by the availability of suitable synthetic probes. Also, while ABPP categorizes the active site in enzymes, it does not measure the functional kinetics of enzymes and therefore can be considered only as an indirect measure of protein function. 

This article proposes a novel approach for localization of a functional enzyme. It forms the central component of the workflow strategy, which has the potential to identify functional biomarkers from natural cellular sources. The proposed method would fill an unmet need for research in drug response and biomarker discovery for investigations in natural cellular source environments. The physiological relevance of working with natural cellular sources is especially significant for discovery, which targets proteins whose function may be altered by post-translational modification, noncovalent regulatory factors or splice variants. Such an approach may help to reconcile data from high-throughput screening of recombinant proteins to natural cellular sources. It is anticipated that select subproteomes will be subjected to downstream characterization by liquid chromatography mass spectrometry (LC-MS), and other suitable identification methods in common use, so as to annotate sequence and structure to function.

 While the term functional proteomicsencompasses a variety of phenotypic descriptions of known or measurablefunctional consequencesincluding cellular response to stimuli [13] and binding interactions [14, 15], etc., the model approach reported herein is limited to characterizing enzyme kinetic properties.

The proposed profiling strategy starts with subfractionation of complex proteomes using SeraFILE [16] (USPTO 20040106131, ProFACT Proteomics, Monmouth Junctions, NJ, USA). This proprietary protein separations platform is configured as a surface library with associated interrogation methods designed to retain bioactivity of the samples. As a result, subproteome pools obtained after SeraFILE separations can be characterized for their enzyme activity properties (e.g., enzyme activity with and without inhibitors, activators, or cosubstrates). Then, a collective functional profile of the original proteome is generated as an integrated profile of the functional properties of the characterized subproteomes. This approach provides multiple data points to characterize and compare samples, thereby increasing the robustness and reliability of analysis. It also allows localization of proteins responsible for sample-specific responses. This profiling method can compare one proteome sample to another (intersample analysis, e,g., tissue type versus tissue type, or normal versus diseased tissue) and can compare different subproteomes of the same complex proteome (intrasample analysis). 

The cyclic nucleotide phosphodiesterases (PDEs) enzyme family has been used in this study as a model class of proteins to demonstrate the proposed strategy. PDEs are enzymes that hydrolyze the second messenger adenosine 3′, 5′-cyclic monophosphate (cAMP) or guanosine 3′, 5′-cyclic monophosphate (cGMP), or both. These small molecules along with other nucleotides, lipids, and ions function as secondary messengers [17]. The second messenger cAMP mediates a wide variety of actions of hormones and neurotransmitters and influences cell growth, differentiation, survival, and inflammatory processes [18]. Class I PDEs (found in protozoa and metazoa) are cAMP specific (PDE4, 7 and 8) or cGMP specific (PDE5, 6 and 9) or can hydrolyze both cAMP and cGMP (PDE1, 2, 3, 10, and 11) [17, 19, 20]. A comprehensive review of PDEs can be found in [17, 19, 21]. 

PDEs are widely acknowledged and explored as drug targets in pulmonary, neurodegenerative, and vascular diseases, and in diabetes, osteoporosis, cancer, rheumatoid arthritis, and depression [22]. Inhibitors of PDE5 and PDE3 are already in clinical use [23], but numerous other PDE inhibitors have not been used for therapeutic purposes due to side effects such as nausea and emesis [24]. The proposed approach to proteomic profiling is guided by the principle that, by discriminating and characterizing PDE variants in natural sources, greater disease-specific therapeutic inhibition/activation can be achieved along with a better understanding of disease pathway dynamics.

This research article demonstrates functional proteomic profiling of cAMP-hydrolyzing phosphodiesterases from bovine and rat brains. Although earlier studies have documented the presence of different types of PDEs in rat and bovine brains, a comprehensive comparative profile of PDE proteomes based on function and content/identity has not been established. It is known that bovine brain exhibits calmodulin-activated PDE activity (PDE1), as well as PDE2, and PDE4 activity [25, 26]. The cAMP hydrolysis activity of PDEs in bovine brain can be stimulated [27] or inhibited [28] by cGMP. Studies on rat brain have identified calmodulin-stimulated PDEs [29–31] (PDE1), as well as PDE4 isoforms [32–34]. 

SeraFILE was first applied for fractionation of each brain homogenate (sample proteome) into subproteomes, in order to reduce the complexity of PDEs in the sample proteome. Then, these subproteomes were interrogated for cAMP hydrolysis activity in the presence and absence of cGMP. cGMP is another substrate of PDEs and is used as a challenge condition in these experiments. The results were compiled into a signature profile of cAMP hydrolysis characteristics of each sample proteome, defined as an integrated profile of characteristics of SeraFILE-generated subproteomes. The hypothesis was that SeraFILE and associated interrogation methods would generate distinct profiles of enzyme catalyzed cAMP hydrolysis-activities from bovine brain and rat brain homogenates because these are different mammalian species. 

## 2. Materials and Methods

### 2.1. SeraFILE Surfaces

 The SeraFILE inventions [16] encompass the surface characteristics and protocols suitable for differential proteomic fractionation. Each surface architecture was designed to have moderate binding capacity and was prepared with Nugel Epoxy (Biotech Support Group Inc., Monmouth Junction, NJ, USA). The epoxy-coated silica was modified by reacting it with different ligands to generate unique surfaces selectivities and was based on the premise that important ligand protein interactions include hydrogen bonds, ionic interactions (salt bridges), hydrophobic interactions and ring structures.  Table 1 illustrates the differences in the properties of the surfaces in the library; however for proprietary protection, details of the surface chemistries remain undisclosed.

An initial screen of 13 surfaces from the library (Table 1) and one underivatized control was performed. Further study was limited to a set of five surfaces (A, B, D, M, and N) from the surface library because the subproteomes obtained from these surfaces had the most distinguishing characteristics (enzyme activity and its response to rolipram/vinpocetine/calmodulin, protein concentrations, and SDS profile, data not shown).

### 2.2. Preparation of Brain Homogenates

 Rat brain homogenate (RBH) and bovine brain homogenate (BBH) were supplied by Lampire Biologicals (Pipersville, PA, USA). Whole bovine or rat brain was homogenized in a prechilled blender using 100 mL of extraction buffer for every 50 g of brain tissue. Extraction buffer for BBH was 0.1 M Tris, 2 mM EDTA, and pH 7.5, and for RBH it was 1 mM EDTA, 10 mM HEPES, and pH 7.4. Each extraction buffer was made with protease inhibitor cocktail (Roche, Indianapolis, IN, U.S.A.). Homogenized brain-buffer mixtures were centrifuged at 4°C, and the supernatant was used for the experiments.

### 2.3. Brain Homogenate Pretreatment (Clarification)

 RBH and BBH samples were mixed with Cleanascite (Biotech Support Group, Monmouth Junction, NJ, U.S.A.) in a 1 : 16 ratio of Cleanascite-to-homogenate, to remove lipids and particulates. Clarified homogenates were obtained by following mixing and centrifugation steps as given in the manufacturer's protocol. 

### 2.4. Sample Separation

 The pretreated homogenates were each subjected to separation by five SeraFILE surfaces (A, B, D, M, and N) [35–38]. For separation of each homogenate, 50 mg of each surface contained in a Spin-X tube (Corning Inc., Corning, NY, U.S.A.) was equilibrated with binding buffer (0.05 M HEPES, 1 mM MgCl_2_, and pH 6.5). Clarified BBH and RBH were diluted in the binding buffer, to pH 6.5-6.6, and 200 *μ*L of each diluted homogenate (load, 1.16 mg of total protein) was added separately to each of the five surfaces, mixed for 10 mins, and then centrifuged. (Note that the total protein amounts used for SeraFILE separations were based on the sensitivity of the cAMP hydrolysis assay used in our experiments for downstream analysis. The SeraFILE methodology is nevertheless amenable to protocols that can use *μ*g amounts of protein loads). The flowthrough was collected as the 1st SeraFILE fraction, represented as subproteomes A1, B1, D1, M1, and N1, from surfaces A, B, D, M, and N, respectively. The proteins bound on the surfaces were eluted with 200 *μ*L of elution buffer (0.05 M HEPES, 1 mM MgCl_2_, 0.5 M NaCl, and pH 8.0) using mixing and centrifugation steps as above. The flow-through collected in this process was the 2nd SeraFILE fraction, represented as subproteomes A2, B2, D2, M2, and N2, from surfaces A, B, D, M, and N, respectively. Mixing steps were performed using a MixMate (Eppendorf, Hauppauge, NY, U.S.A.) at 1150 rpm following an initial pulse of mixing on a vortex mixer. Centrifugation steps were performed using a tabletop centrifuge at 16873 rcf for 3 mins. Each brain homogenate, bovine and rat, was used for separations in triplicates. 

### 2.5. cAMP Hydrolysis Activity Assays and Protein Assays

 Activity of cAMP hydrolysis in each subproteome was measured using a real-time kinetic assay [39, 40]. This assay links cAMP hydrolysis to NADH oxidation using coupling enzymes (adenylate kinase, pyruvate kinase, and lactate dehydrogenase), and NADH loss can be measured at 340 nm. For each assay, a mixture of reaction buffer and coupling enzymes was equilibrated at room temperature for 16 mins (stage I). Then, subproteomes were each individually added to the reaction mix, and loss in absorbance was measured for 16 mins (stage II). Finally, substrate cAMP or a mix of cAMP and cGMP was added to the assay, and the loss in absorbance was measured as above (stage III). Final concentrations of assay components were as follows: 9 mM MgCl_2_, 0.46 mM CaCl_2_, 46 mM KCl, 46 mM HEPES, 1 mM phosphoenolpyruvate, 46 *μ*M ATP, 0.4 *μ*M NADH, 50 *μ*M cAMP, 0.8 units pyruvate kinase, 4 units lactate dehydrogenase, and 0.06 units adenylate kinase (Sigma, St. Louis, MO, U.S.A.) with 6.25 *μ*L of each enzyme sample. The cAMP hydrolysis activity of each sample was measured as the basal activity (in the absence of cGMP) and as challenged activity (in presence of 25 *μ*M cGMP or 50 *μ*M cGMP). Final volume of the assay was 0.1 mL. Volume-normalized enzyme assays were performed on each replicate subproteomes in a 96-well format using a Multiskan MMC346 plate reader (Thermo Scientific, Hudson, NH, U.S.A.). 

To measure cAMP hydrolysis activity in the unfractionated brain homogenates, the clarified homogenates were diluted with binding buffer to obtain 5-6 dilutions of each homogenate, which were then used for the assays as described above. 

Protein content of all RBH and BBH proteomes and subproteomes was measured using a BCA assay kit (Pierce, Rockford, IL, U.S.A.). Replicate subproteomes were pooled before protein analysis.

### 2.6. Calculations

(a) The cAMP hydrolysis activity of each sample was calculated as follows:

(1) Enzyme  activity  (nmoles mL−1 min⁡−1)   =Path  length  correction  factor×corrected  PDE  rate  (min⁡−1)×reaction  volume  (mL)×dilution  factorMolar  absorption  coeffcient  (M−1 cm−1)×sample  volume  (mL),

where corrected PDE rate is ΔA_340 nm_(min⁡^−1^) of stage III − ΔA_340 nm_(min⁡^−1^) stage II, molar absorption coefficient is 1.25 × 10^4^ M^−1^  cm^−1^, dilution factor is 1, and path length correction (to 10 mm) is 3.16.

(b) The % change in cAMP hydrolysis activity of each sample was calculated as follows:

(2) %  Change  in  cAMP  hydrolysis  activity=(Challenged  enzyme  activity−Basal  enzyme  activity)×100Basal  enzyme  activity,

where challenged enzyme activity is cAMP hydrolysis activity in presence of cGMP, and basal enzyme activity is cAMP hydrolysis activity in absence of cGMP.

## 3. Results and Discussion

### 3.1. Sample Pretreatment

 Cleanascite [41–44], a solid-phase, nonionic adsorbent for lipid removal, significantly improved the clarity of brain homogenates and eliminated clogging of the surfaces during the SeraFILE process. A 1 : 16 ratio of Cleanascite to untreated BBH gave optimal results, with minimum loss of cAMP hydrolysis activity. Consequently, the same ratio of Cleanascite to brain homogenate was used for RBH clarification.

### 3.2. cAMP Hydrolysis Activity in the Sample Proteomes

 Enzyme activity and protein analysis of the unfractionated brain homogenates showed that the mean specific activity of clarified RBH and BBH was comparable, between 8 and 8.8 units/mg, measured at 50 *μ*M cAMP concentration.

### 3.3. Effect of cGMP on cAMP Hydrolysis of Unfractionated Brain Homogenates

**Figure 1:**
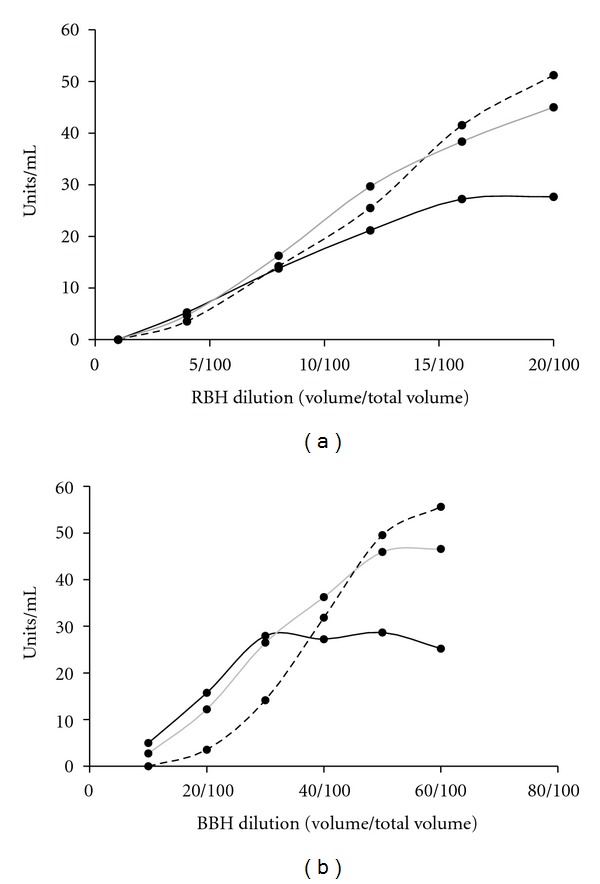
The cAMP hydrolysis activity in clarified rat brain homogenate (RBH), (a), and bovine brain homogenate (BBH), (b), proteomes. The cAMP hydrolysis activity was measured by using dilutions of the clarified homogenates in the absence of cGMP (solid black) or in presence of 25 *μ*M cGMP (solid gray) or 50 *μ*M cGMP (dashes).

**Figure 2:**
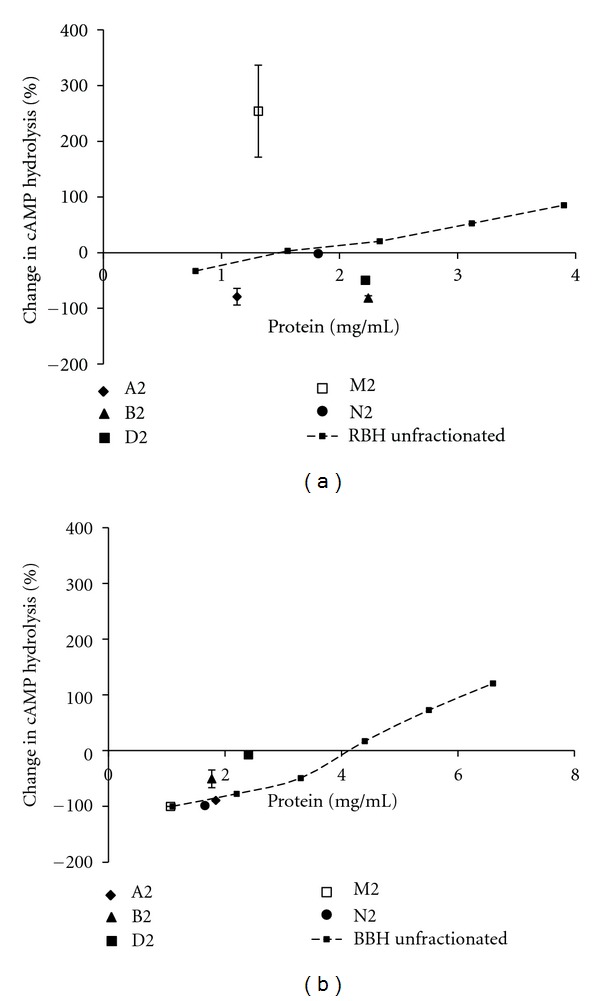
Relationship between change in cAMP hydrolysis activity and protein content of the unfractionated brain homogenates and SeraFILE-generated subproteomes. The figure shows change in cAMP hydrolysis of rat brain homogenate (RBH) and generated subproteomes, (a), and bovine brain homogenate (BBH) and generated subproteomes, (b). *X-*axis represents protein concentration. *Y-*axis represents percentage change in cAMP hydrolysis activity due to the challenge of 50 *μ*M cGMP, as compared to basal cAMP hydrolysis activity. A2, B2, D2, M2, and N2 represent subproteomes from the homogenates.

 A comparison of the basal and challenged cAMP hydrolysis activities in the dilutions of each homogenate is shown in Figure 1. As expected, increase in activity (basal and cGMP challenged) was observed with increasing concentration of clarified homogenates. The comparison also shows that at relatively lower concentrations of the homogenates, cGMP inhibited cAMP hydrolysis, while, at higher concentrations, cAMP hydrolysis activity increased. In addition, the change in cAMP hydrolysis activity was more pronounced in the presence of 50 *μ*M cGMP than 25 *μ*M cGMP. Specifically, at 50 *μ*M cGMP (Figures 2(a) and 2(b)), the change from inhibition to activation of cAMP hydrolysis activity occurred above ~1.5 mg/mL protein in RBH and above 4 mg/mL protein in BBH proteomes. Thus, it is a characteristic in the PDEs of RBH and BBH, that the effect of cGMP on cAMP hydrolysis is a function both of the concentration of the homogenate and of the concentration of cGMP.

### 3.4. SeraFILE-Derived Subproteomes and Generation of Enzyme Activity Profiles

 Buffer-diluted, protein-normalized, and clarified RBH and BBH samples were used for separations. Each subproteome obtained was analyzed for protein content and cAMP hydrolysis activity under basal and cGMP-challenged conditions, and then the change in cAMP hydrolysis activity was calculated.

To ensure that the observed properties of the subproteomes were not an effect of the dilution of the sample proteome, the properties of RBH and BBH proteomes and their respective SeraFILE subproteomes were compared (Figures 2(a) and 2(b)). The data in Figure 2(a) show that not all RBH subproteomes follow the activity versus protein content relationship of the RBH proteome. Similar observations were made with respect to BBH (Figure 2(b)). These outliers indicate that SeraFILE produces differential subproteomes. Data show that some subproteomes do share the activity versus protein content relationship of the sample proteome, likely indicating a comparable distribution of cAMP hydrolyzing PDEs to total proteins. 

**Figure 3:**
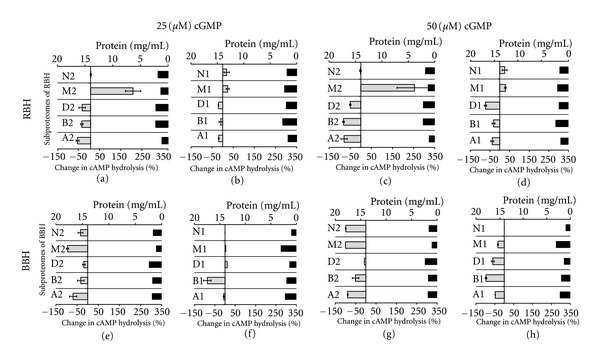
Comparison of functional profiles of rat brain homogenate (RBH) and bovine brain homogenate (BBH). Figure shows percentage change in cAMP hydrolysis activity in each subproteome of RBH ((a)–(d)) and BBH ((e)–(h)) due to cGMP challenge of 25 *μ*M or 50 *μ*M. In each panel, the primary *X*-axis represents the change in cAMP hydrolysis due to cGMP, the secondary *X*-axis represents protein concentration, and the *Y*-axis represents subproteomes. The pair of subproteomes A1 and A2 (and similarly others) was derived sequentially from the same surface in the library as described in the protocol. Grey bars represent mean percent change (*n* = 3), in cAMP hydrolysis of each subproteome due to presence of cGMP. Error bars represent (±1) std. Black bars represent protein concentration of each subproteome.

To generate an intersample functional proteomic profile of RBH and BBH proteomes, the change in cAMP hydrolysis activity of each subproteome due to cGMP challenge was calculated and plotted as shown in Figure 3. A functional proteomic profile of the brain homogenates in these experiments is defined by the collective response of individual SeraFILE subproteomes to cGMP challenge. A comparison between the functional profiles of the two homogenates (Figure 3) shows that, overall, these two profiles have a similar pattern (i.e., % change in cAMP hydrolysis is similar, positive or negative, in comparable fractions of the two homogenates). However, a major difference is found in subproteome M2 of RBH and BBH (refer to Figure 3(a) versus Figure 3(e), and Figure 3(c) versus Figure 3(g). Subproteome M2 of RBH shows over 190% increase in cAMP hydrolysis in the presence of cGMP (both 25 *μ*M and 50 *μ*M), while subproteome M2 of BBH shows over 90% decrease in cAMP hydrolysis in the presence of cGMP (both 25 *μ*M and 50 *μ*M). Thus, subproteome M2 is a differentiating feature of this inter-sample analysis and therefore can be used for further sample characterization. 

 These model data demonstrate that our proposed methods of protein separation generate subproteomes that are sufficiently differentiated for intersample functional analysis. As a result, these methods can be potentially applied to effectively differentiate functional properties of complex proteomes and can be used to localize subset of proteins attributable to sample-specific responses. The localized proteins can then be used for further analysis, characterization, and subsequent MS identification (gene sequence annotation/reconciliation).

SeraFILE separations use mild-to-moderate elution conditions with buffers like phosphate or HEPES that are commonly used in the laboratory. In addition, the solid-phase surface (50 *μ* derivatized silica) can be easily removed by filtration. Thus SeraFILE separations methods do not introduce substances like urea or SDS that may restrict downstream compatibility with existing reporting assay and LC-MS detection methodologies [45]. Therefore, the proposed methodology is considered to have a broad scope of applicability within the pathway to identification and can be potentially applied to profile narrowly defined therapeutically important classes of enzymes such as Kinases or cyclic nucleotide phosphodiesterases. 

Another important characteristic of SeraFILE separations methodology is its reproducibility at different protein loads. In separate experiments, surface separation of 0.25 mg to 1 mg protein per 50 mg of surface was shown to have only 10% variation (data not shown, [35]). The reproducibility in sample separation can be significant for heterogeneous samples of clinical origin. 

In addition to separations, SeraFILE can also be applied for enrichment of proteins. Incremental increase in pH was applied for enrichment of alkaline phosphatase (data not shown, [35]) with an enrichment factor up to 20X.

The applications of SeraFILE separations can be based on two basic types of sample and data analysis of (i) intersample analysis whereby samples such as tissues, cellular models, or biofluids are compared and contrasted and (ii) intrasample analyses, or differential analysis within a sample whereby the subproteomes are monitored with respect to a challenging modulation condition such as in drug response profiling. It is envisioned that these will complement one another for personalized medicine applications.

Inter-sample analysis of complex proteomes, as demonstrated, potentially applies to disease and nondisease comparisons, to identify differences in samples by compartmentalizing the most distinctive subproteomes associated with disease. Deeper characterization of these fractions with enrichment (e.g., with pH optimization of SeraFILE separations, or with conventional separations 2DE or HPLC), followed by LC-MS analyses, can help identify prospective biomarkers. It is important to recognize that any biomarker panel selected in this context would require more characterization, with larger sample sets and statistical validation.

Intra-sample analysis, on the other hand, can be used to catalogue or index the effects of functional modulation of the daughter subproteomes. This will be especially valuable for establishing localized panels of proteins that are responsive to modulation with drug compounds, with the same caveats as the aforementioned inter-sample analyses. 

The two data analysis strategies, profiling between samples, and cataloging within samples, are complementary insofar as molecular profiles that characterize and compartmentalize drug-responsive proteins from complex mixtures, can potentially, through coincident iterations with disease profiling, create a bulls-eye effect for drug repurposing. 

We envision that, for the drug development industry, the proposed methods for localizing proteins with known functional attributes offer new resources for biomarker discovery, complementing conventional methods of identification and sequence annotation. For drug compounds, a challenge/response method, as described, can help address the problems of drug promiscuity and discern the subtleties of protein attributes; when the same or similar underlying sequences, have multiple conformations and functions, and when different sequences sometimes perform the same or similar function. 

As a way to begin sifting through these biological complexities, a more efficient method to characterize protein function and corresponding modulation is now possible. Starting with the enrichment of prospective functional biomarkers in localized subproteomes, we suggest that structural and sequence relationships can be determined. Such an approach has the potential to provide new and useful service to biomarker discovery and personalized medicine. 

## Figures and Tables

**Table 1:** Mixed-mode properties of SeraFILE surface structures^a^. Table shows potential numbers of hydrogen bond donor/acceptor groups, numbers of cationic/anionic groups, and number of ring structures in the surface ligands, along with relative hydrophobicity of the ligands.

	Surface	Hydrogen bond		Cationic groups	Anionic groups	Relative hydrophobicity^b^	Rings
		Donor groups	Acceptor groups				
Surfaces used in the study	A	1	2		1	2	
	B	1	6		3	2	1
	D	1	4		2	2	1
	M	Multipolymer				1	
	N			1		1	

Surfaces initially screened, but not used in the study	PN	3		3		3	1
	E			Multipolymer		3	
	AP				Multipolymer	1	
	AM	1	2	1		1	
	S				Multipolymer	5	Multipolymer
	F			1		4	
	C	1	2		1	5	1
	PL			1		3	
	PA	1	1		1	4	1
	PC			1		5	1

^a^In cases of polymers, only predominant effect is considered.

^b^Scale 1–5: low-high.
